# Novel Food-Based Product Communication: A Neurophysiological Study

**DOI:** 10.3390/nu12072092

**Published:** 2020-07-15

**Authors:** Vincenzo Russo, Giulia Songa, Laura Emma Milani Marin, Claudia Maria Balzaretti, Doriana Eurosia Angela Tedesco

**Affiliations:** 1 Department of Business, Law, Economics and Consumption “Carlo A. Ricciardi”, Iulm University, 20143 Milan, Italy; vincenzo.russo@iulm.it (V.R.); giulia.songa@gmail.com (G.S.); 2 Centre of Research of Neuromarketing “Behavior and Brain Lab”, Iulm University, 20143 Milan, Italy; 3 Department of Veterinary Science for Health, Animal Production and Food Production, University of Milan, 20133 Milan, Italy; claudia.balzaretti@unimi.it; 4 Department of Environmental Science and Policy, University of Milan, 20133 Milan, Italy; doriana.tedesco@unimi.it

**Keywords:** food choice, novel food label, neurophysiology, consumer psychology

## Abstract

The steady increase in the global food demand requires alternative sources. Food sources from invertebrates could be a viable alternative. Despite a growing interest in terrestrial invertebrates as novel food, Western consumers have to cope with fears and taboos. This research aims to investigate possible communication strategies of novel food through labels. To understand the complexity underlying food choice and novel food attitude, two studies were carried out. In Study 1, the main drivers in the food decision-making process were identified. Based on these results, in Study 2, two different food labels for crackers made with earthworm flour were designed. Applying a neurophysiological approach, we measured participants’ neuropsychophysiological activation and behavioural response while watching food labels. A video on nutritional and ecological issues was shown to consumers to reduce aversion towards earthworms as food. The results in Study 1 indicate health and sensory dimensions as the major drivers in food choice. The data of Study 2 supported the effectiveness of the statement about nutritional qualities of the products on male participants, who tend to have a more positive reaction than female participants toward the novel product made with earthworm flour when the label’s claim focuses on nutritional advantages. Limitations and practical implications are discussed.

## 1. Introduction

It is estimated that in 2050, population growth will increase the global food demand by up to 70% compared with the present demand [1]. The steady growth of the population and the contextual diminishing resources require a paradigm shift in food consumption, especially for the Western population. Introducing more sustainable food sources, such as insects, will ameliorate the health of both the Earth and of human beings, in addition to supporting socio-economic development.

In January 2018, the European Regulation on novel food (2015/2283) went into effect in all European countries, bringing new foods and ingredients (Regulation EU 2015/2283–Article 3). Among them are terrestrial invertebrates (e.g. insects and earthworms). Therefore, it is useful to understand how to encourage acceptance of novel food among Italian consumers. Despite a growing interest in non-traditional and sustainable foods, Italian consumers still experience fears and taboos [2]. Hence, it is necessary to increase awareness among people in order to change attitudes and values towards novel food [3]. Communication plays a crucial role in information dissemination and food habit changes [4]. In particular, it is important to identify a better way to communicate information through labels [5]. Information dissemination and taste play a guiding role in modifying inclinations and attitudes towards novel food [6]. However, to the best of our knowledge, only one previous study explored the achievability of such change with a multi-method study combining a preliminary quantitative phase with a subsequent neurophysiological study [7].

In order to build an effective communication campaign, it is necessary to know the underlying purchasing food motivations. Food decision-making is a complex process involving psychological, social, cultural, economic and biological factors [8]. The Food Choice Questionnaire [9] is one of the most widely used models to investigate food motivations. Understanding motivational differences in food choice among consumers could be crucial to identifying those aspects involved in a transitional process through which novel food is accepted. Motivations concern both individual and social dimensions [10]. A recent research study carried out in Italy [6] revealed how communication about both individual and social benefits of eating novel food increases the intention of eating this food. Furthermore, previous knowledge of eating insects seems to have a positive impact on the taste rating, [11] and consumers are more likely to accept and eat novel food if it is presented in a hybrid form [6,7,8,9,10,11,12]. Moreover, the product’s name can influence consumers; using familiar names and the names of individual species may be a good communication strategy to diminish neophobia [13].

In addition to the previously mentioned instance, consumer food purchasing motivations are influenced by extrinsic product features such as labelling and packaging, which are two of the prime ways to communicate with consumers. Therefore, the product design is a crucial element to determine the success of a product [14] since expectations, arising from packaging information, can influence the perceived quality of a product [15]. Moreover, food labels may influence consumers’ willingness to buy if they provide convincing information [16] and evoke positive emotional reactions [17].

Although people are still not disposed towards this novel food, some positive findings on acceptance have emerged [18]. Because the introduction of novel food is a delicate topic for Italian consumers, a suitable way to assess individual emotional reaction to this type of novel food is needed. Following the “dual process theories”, the decision-making process follows intuitive (implicit) and deliberate (explicit) paths [19,20] that can interact together. Decision making involves the use of both automatic and controlled processes [21]. The role of the context is also added into the model. Indeed, depending on the specific context and the person’s previous experience, the decision may differ. In fact, if a person has to make a decision involving different motives (e.g., rational vs emotional), he could follow rational or emotional motives depending on the situation and the individual’s preferences. Other authors assert that intuitive processes and analytical processes interact together [22].

Thus, consumers’ choices can be driven by a deliberative reasoning or by a temporary emotion [23]. These different dimensions should be assessed with different measurements: direct techniques consisting of explicit questions to the subjects are suitable to measure the conscious part of opinions and attitudes [24]. Indirect techniques objectively measure the spontaneous part of the emotional reaction. Emotions have a physiological substrate, and thus specific variations in body parameters reflect emotions [25]. The analysis of neuropsychophysiological signals allows for the understanding of the mental processes that occur below the threshold of conscious awareness [26,27] because this type of measurement is continuous and objective [28].

The aim of the present research was to investigate communication strategies through labels on food packaging. The first objective was to understand the main drivers of food purchasing decisions. Based on the food choice literature [9,10,11,12,13,14,15,16,17,18,19,20,21,22,23,24,25,26,27,28,29], the main drivers in the decisional process were identified (Study 1). The second objective was to understand the optimal communication strategy to promote crackers made with earthworm flour. Based on the results of Study 1, in Study 2, two different food labels for crackers made with earthworm flour were designed. This label allowed us to present novel food with a familiar shape and name. The effectiveness of the two labels was compared in terms of their ability to evoke a positive emotional response. To assess the emotional reaction of consumers, a neurophysiological approach was used to measure participants’ psychophysiological and neurological activation (skin conductance, electroencephalography - EEG) and behavioural response (distance from the screen) while looking at food labels. In order to reduce aversion towards earthworms, a video on nutritional and ecological issues was displayed to consumers.

In the present research we refer to the new Regulation (EU) 2015/2283 on novel foods. We only focused on an earthworm taxon in Study 2 since the macro research project is about the “Bioconversion of fruit and vegetable waste to earthworm meal as novel food source” (see Acknowledgments).

## 2. Materials and Methods

### 2.1. Study 1—Food Choice Questionnaire

Subjects and Procedure: In this study, 1285 respondents (female 647) aged between 18 and 29 years were administered a self-reported questionnaire. Students at two Italian universities, who are in charge of shopping (or who share this responsibility in the household) voluntarily participated in the research. We chose a specific target, young adults, since they are future consumers and food decision makers.

Items on food choice were drawn from the Food Choice Questionnaire [9], with the addition of some items. The questions were related to food eaten on a typical day. For the purposes of the present research, the original FCQ was translated into Italian and translated back into English by two independent translators. The items were answered on a seven-point interval scale ranging from ‘not important at all’ (=1) to ‘extremely important’ (=7). After the translation into Italian, the questionnaire was pre-tested through interviews with ten students and a wider pilot administration of sixty students. Through the results and the literature on food quality certification [30,31], branding [32,33] and ethical food choice motives [34,35], some items were generated, and others were eliminated. Six new items were added: “Is a well-known brand”, “Provides all the nutrients required”, “Has a food quality certification (e.g., PDO, PGI etc.)”, “Is organic”, “Is locally produced/cultivated” and “Is a fair-trade product”.

### 2.2. Study 2—Neuro-Psychophysiological Activation and Behavioural Response

For the second study, two different food labels were created, identical in terms of design and product name (the Italian term corresponding to “earthworm flour”), with two different claims. The first claim was related to the product healthfulness stating that it is gluten-free and rich in protea. The second described the possibility to enhance the flavour of daily food. The two labels were compared in terms of effectiveness, namely increased acceptability, in promoting the product. Furthermore, a comparison was made between genders, in order to understand if there are any differences in terms of communication strategy. The lettering “earthworm flour” was deliberately designed to produce an unpleasant effect on respondents.

#### 2.2.1. Participants

Forty-four participants (24 females) aged between 19 and 29 years (M = 21.22, SD = 3.43) were recruited from a list of research volunteers at two Italian universities in order to have a sample with the same characteristics of Study 1. All participants were experimentally naïve. All of the participants gave their informed consent prior to taking part in the study.

The sample was split in two groups. For one group, the “earthworm flour” label showed the claim related to the healthfulness of the product, and for the other group, the claim was related to the taste.

#### 2.2.2. Design and Procedure

Each participant conducted the experiment individually in a quiet room with consistent temperature and lighting. Upon arrival, participants were seated 60 cm away from the computer display of the SMI-RED250 eye movement recording system. After a brief explanation, participants were informed that their privacy would be guaranteed by the usage of a code, instead of their name, to identify them. They wore the equipment for the recording of neuro-psychophysiological signals (EEG headset and skin conduction sensors) and a 5-point calibration was carried out using SMI iViewX software before starting the recording of eye movements. First, the signals were recorded in a state of relaxation (baseline) in order to have a benchmark to measure the activation due to the experimental stimuli. Participants were then presented with crackers (with different labels) in different conditions. 

In the first phase (blind taste), participants tasted the food without any information. Then, they watched the image of the products with the label providing the information before the second taste. These products were crackers made with grain flour and the same crackers labelled, “Made with earthworm flour.” The images were presented in a balanced order to avoid effects of order. After that, participants were exposed to a video explaining the advantages of eating alternative and more sustainable food sources. The video presented novel food as “the food of the future,” with a narrator describing the product as excellent in nutritional value and for environmental sustainability. There was an attempt to make insects and other terrestrial invertebrates as food less strange for the consumer, comparing them to products such as soya and kiwi (introduced in Western consumption relatively recently) and showing images of smiling people eating them, sample recipes, young Italians organizing workshops, and people cooking with the products.

**Figure 1:**
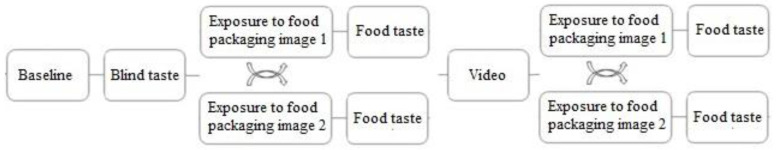
Schematic of the experimental procedure. Food packaging 1 refers to crackers with a label stating that the product is made from grain flour, Food packaging 2 refers to crackers with a label stating that the product is made from earthworm flour (with a different claim for the two groups). The two products were presented in random order.

Subsequently, they tasted the crackers again while watching an image of the product with the label. The two products (labelled “earthworm flour”/“grain crackers”) were presented in random order. After each phase, participants were asked to express their feeling toward the crackers they had seen or tasted using the Self-Assessment Manikin (SAM) valence scale [36], which is a “culture-free” and “language-free” scale [37]. A schematic of the experimental design is shown in Figure 1. At the end of the experiment, subjects were asked to indicate the food products they thought they had tasted. All the subjects thought they had tasted crackers made with grain flour and earthworm flour. Afterward, a researcher explained to the participants that all the crackers were made with grain flour clarifying the aims of the experiment.

During the experiment, participants’ eye movements and neuro-psychophysiological signals were recorded. 

#### 2.2.3. Stimuli

**Figure 2:**
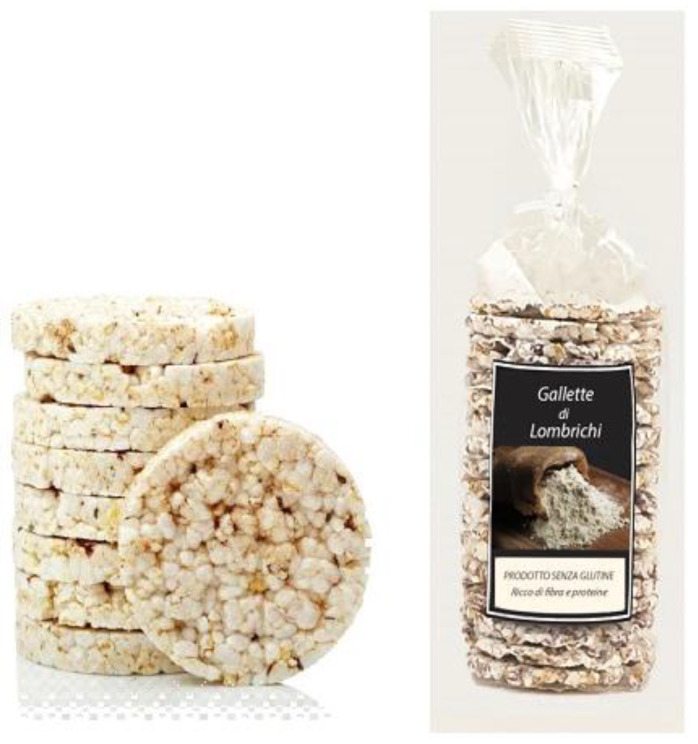
Examples of crackers and cracker packaging images.

The stimuli consisted of crackers of the same brand, all made from grain flour and images of the crackers with mock labels (indicating earthworm flour) (Figure 2):

- Label stating that the product is made with grain flour;
- Label stating that the product is made with earthworm flour, version 1 (healthfulness information, group 1);
- Label stating that the product is made with earthworm flour, version 2 (tastiness information, group 2).

### 2.3. Data Analysis

Confirmatory factor analysis was performed (SPSS 22.0) to validate the item suggested in the FCQ [9]. Exploratory factor analysis was performed (SPSS 22.0) for new measures. A preliminary factor analyses on 38 items (6 new and 32 original FCQ items) was conducted [32]. The item “Is like the food I ate when I was a child” on familiarity factor was excluded because it decreased the internal consistency, which was lower than the threshold value for satisfactory scales [38]. As elsewhere, convenience and availability were split into two different dimensions [39]. The item “Is a well-known brand” converged the latent variable familiarity; “Provides all the nutrients required” converged the latent variable health; “Has a food quality certification (PDO, PGI or TSG.)”, “Is organic”, “Is locally produced/cultivated” and “Is a fair-trade product” converged the latent variable ethical concern. Meanwhile, “Is approved politically” and “Has country of origin marked” were excluded from ethical concerns because they decreased the internal consistency, which was lower than the threshold value for satisfactory scales [38]. For the same reason, the item “Is good value for money” was excluded from price factor.

The parameters of neuro- and psychophysiological activation were standardized with the baseline values to measure the variation from a neutral activation due to the specific stimulation [40]. An increase in skin conductance is an index of arousal, indicating emotional response intensity [41]. Skin conductance is the electrical conductivity of the skin when an external voltage of constant voltage is applied. Sweat contains a conductive saline solution which causes a decrease in the resistance to the passage of the current and therefore an increase in the skin’s ability to conduct electric current. This variation is measured through a device with two branches placed on the fingers or on the palms of the non-dominant hand. An increase in conductivity, due to a greater production of sweat attributable to a greater activation of the sweat glands controlled by the sympathetic nervous system through the hypothalamus, is an index of arousal (activation) of the subject [41]. The duration and amplitude of the signal are influenced by the emotional value of the stimulus [42,43]. In fact, the sweat glands respond better to psychological stimulations than temperature changes [44].

The EEG signal was used to isolate the alpha band to compute an index of frontal asymmetry. Based on the principle of the lateralization of brain functions, there is a selective activation of the left (right) part of the cerebral cortex in response to positive (negative) stimuli [45]. The left part of the frontal cortex is part of the cerebral circuit involved in the experience of positive emotions, which leads to a tendency of approach toward stimuli perceived as desirable. The corresponding area on the right is a component of the circuit involved in the processing of negative emotion and in defensive withdrawal [46]. A decrease in the alpha power (“alpha inhibition”) in a particular brain region indicates a greater cortical activation [47]. The asymmetry in the alpha band (FAA, frontal alpha asymmetry) is the difference between the power in the frequency band in the left and right frontal hemispheres, respectively. Thus, a positive value of the FAA index indicates a greater activation of left than of the right hemisphere.

From the eye tracking data, we computed an index of distance from the screen. People have a tendency to decrease the physical distance between them and a stimulus when they appreciate it, while an increase in distance is an index of avoidance regarding the stimulus [48]. Approaching positive objects and avoiding negative ones is a central requirement in motivation [49]. When the object is the point of reference (as in this case, in which the participants could not move the image that is presented on the screen), the self varies its distance from the object by moving toward it (approach) or away from it (avoidance). Approach motions result in a decrease in distance between oneself and the object, whereas avoidance motions result in an increase in that distance [48]. 

## 3. Results

### 3.1. Results Study 1—Food Choice Questionnaire

Table 1 shows the scales, items, mean/SD, Cronbach’s alpha and standardised factor loading of the Food Choice Questionnaire.

Single item standardised factor loadings were all highly significant with values ranging from 0.56 to 0.92. The internal consistency of constructs, which was assessed by Cronbach’s alpha (SPSS 22.0), was good (0.72) and the internal consistency for each construct ranged from 0.66 to 0.87 indicating moderate to good reliability. At the individual item level, the highest weight was allocated to motives “Tastes good” (6.48), “Makes me feel good” (6.22), “Keeps me healthy” (6.16) and “Provides all the nutrients required” (6.03). Table 1 shows the importance of each food choice factor. The sensory (5.53) factor was the most important, followed by the health (5.38). The least important factors were familiarity (3.92) and weight control (4.17).

### 3.2. Results Study 2—Neuro-Psychophysiological Activation and Behavioural Response

#### 3.2.1. Effect of Labels

**Figure 3:**
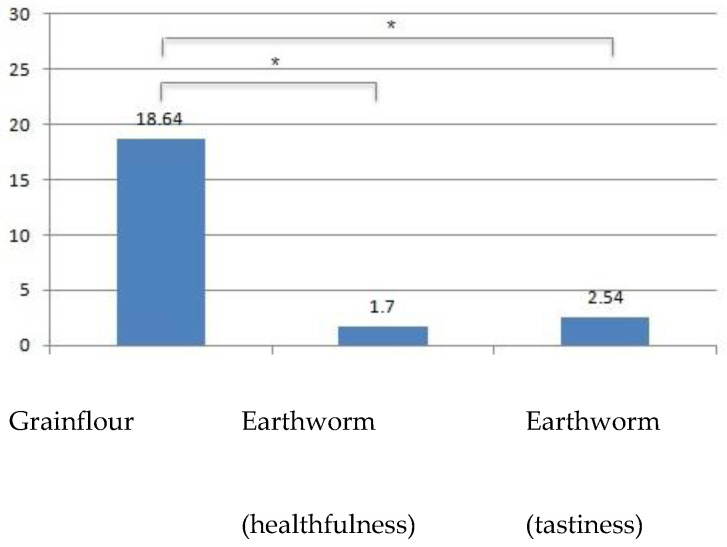
Comparison of participants’ skin conductance (y = microSiemens) response while watching labels. The * in Figure 3 means “statistically significant difference”.

1. Skin conductance. A one-way ANOVA showed that effect of the label on the skin conductance level was significant (F[2,117] = 16.15; p < 0.001). A pairwise comparison indicated that there was a statistically significant difference between the “grain flour” and the “earthworm flour” labels both for the tastiness claim (*p* < 0.001) and the healthfulness claim (*p* < 0.001). The difference between the two “earthworm flour” labels with the different claims was not significant. Figure 3 shows the comparison among the labels.
2. Distance from the screen. An ANOVA was run with the between-subject components: the gender of the participants and the claim (focused on healthfulness/taste). The dependent variable was participant’s distance from the screen while watching images of earthworm flour products. The results show a significant main effect of both claims (F[1,608] = 140.27, *p* < 0.001), and gender (F[1,608] = 193.84, *p* < 0.001). In particular, the claim focused on healthfulness led to a stronger approach response (mdistance = 45.4 cm; SDdistance = 6.6) in comparison with the claim focused on taste (mdistance = 48.52 cm; SDdistance = 4.2). Female participants tended to keep a higher distance from the screen (mdistance = 48.9 cm; SDdistance = 4.6) than did males (mdistance = 45 cm; SDdistance = 6.1) while watching the image of the crackers made with earthworm flour, independent of the claim. Moreover, a significant interaction between gender and claim was found (F[1,608] = 292.06, *p* < 0.001), indicating that the effect of the claim on the response variable was not the same for males and females. A pairwise comparison indicated that there were no gender differences in the response to the product with the claim about tastiness, while distance from the screen is lower for males (Mdistance = 41.2 cm, SDdistance = 6.7) than for females (Mdistance = 49.6 cm, SDdistance = 4.6) when participants were watching the product with the claim about healthfulness.
3. EEG data. As for the distance from the screen index, an ANOVA was run to compare the neurophysiological response (FAA index) of the subjects while watching the images of earthworm flour crackers, based on participants’ gender and on the claim. The results show no significant effect, neither of gender nor claim, while a significant interaction effect between the two variables was found (F[1,12] = 1.04; *p* < 0.05). This means that the effect of the claim on the FAA response was not the same for males and females. A pairwise comparison indicated that there was a statistically significant difference between males (MFAA = 0.18, SDFAA = 0.65) and females (MFAA = −0.37, SDFAA = 1.1) when participants watched the image of the product with the claim about healthfulness. The difference between males and females was not significant for the product with the claim about tastiness.

#### 3.2.2. Effect of the Video

We wanted to verify whether the video had an effect on participants’ explicit (aware) and/or implicit (unaware) perceptions of crackers made with earthworm flour.

1. Explicit evaluation. An ANOVA was run to compare the rating (SAM scale) of the “earthworm” images based on the claim (healthfulness/tastiness) and the experimental phase (before/after the video). The results show no significant effect on explicit evaluation, neither of the experimental phase nor the interaction between the claim and the phase. The difference between the evaluation of the crackers based on the claim was close to statistical significance (mean_healthiness_ = 3.1, mean_tastiness_ = 2.6; F = 3.85; *p* = 0.054).
2. Implicit reaction. Two ANOVA (2 × 2) analyses on the FAA index and distance from the screen were run to compare the neurophysiological and behavioural response of the subjects while watching the images of crackers made with earthworm flour based on the claim and the experimental phase. The results show a significant effect of the interaction between the claim and the experimental phase for the FAA index (F[1,12] = 5.6; *p* < 0.05). This means that the effect of the claim on the FAA response was not the same before and after the watching of the video. A pairwise comparison indicated that there was a statistically significant difference between participants’ responses to the “earthworm” crackers image with the claim about taste before and after the video. In particular, participants had a higher FAA index when exposed to the images of earthworm crackers after (M = 0.005, SD = 0.06) than before the video (M = −0.026, SD = 0.01) only for the claim about tastiness (t = −2.75, *p*-value < 0.01).

The results regarding the distance from the screen are consistent with the ones about FAA index. Indeed, the results show a significant effect of the interaction between the claim and the experimental phase (F = 155.52, *p*-value < 0.01). A pairwise comparison indicated that, when exposed to the “earthworm” image with the claim about taste after the watching of the video, participants were closer to the screen (M = 45 cm; SD = 6.4) than when exposed to the same stimuli before watching the video (M = 52 cm; SD = 6.7; t = −18.48, *p*-value < 0.01).

## 4. Discussion

The aim of the present research is to investigate communication strategies through labels on food packaging.

The objective of the Study 1 is to assess the main food choice motives among young students in Italy. From the analyses, it emerges that sensory and health factors are key drivers during food purchasing. According to the literature on novel food, these are the most investigated factors in research studying consumers’ predisposition to eating novel food [50]; moreover, sensory dimension is a relevant element in developing acceptability and willingness to try novel food [10].

At the individual item level, the average for “Makes me feel good” (6.22) and “Keeps me healthy” (6.16) are the highest, confirming the importance of the hedonic dimension and the attention to health in food choice among consumers. Importance to “Tastes good” (6.48) corroborates the relevance of this sense in food consumption. In particular, taste-sensitive consumers with high levels of education can be considered trendsetters in the consumption of novel food [12]. Familiarity seems not to play a relevant role, and this could be a positive aspect, denoting young consumers’ openness to novel food. In the first study, the results indicate “sensory” and “health” as the main drivers of food choice.

Based on this result, in the second study, two strategies of communication through food labels on packaging were compared using behavioural and neuropsychophysiologial parameters to measure consumers’ emotional reaction to food labels before and after watching an informative video. Participants showed a different response to crackers based on the origin indicated on the label, with a higher emotional reaction to grain flour crackers than to the ones labelled as made with earthworm flour. This result is consistent with the idea that food made with earthworm flour may meet resistance from Italian consumers.

A significant difference was found between the two communication strategies, as the claim about healthfulness provoked a higher approach to the product, especially for males. In general, females showed a greater refusal to this novel food. A significant effect of the video with the information about the product was found, as it diminished the tendency to avoid the crackers made with earthworm flour. Overall, the results highlight that communication can strongly influence consumers’ reaction to novel food and suggest that the most effective strategy relies on healthfulness. Moreover, the results indicate that there are important gender differences in food acceptance to be taken into account when planning a communication strategy. These results have a practical implication for label design based on the specific target and highlight the importance of both self-reported measures and neuro-psychophysiological measurements to obtain complete information about consumers’ emotional and cognitive responses. In fact, these two responses are extremely closely interrelated, and extrinsic information, such as labels, is processed in the brain regions related to emotions [51].

Despite the benefits deriving from the new protein sources, Italian consumers have concerns and worries about it. A decrease in novel food refusal can be reached, giving information about the nutritional and health properties of products, since knowledge influences the perceived quality. To promote the acceptance of this food, an effective communication through food labels that increases awareness is a key strategy. The present research focuses on students because they are future consumers and food decision makers.

With reference to limitations, the first study considered only young adults. We were interested in understanding the most appealing aspects, according to young consumers, of novel food. In fact, young consumers are generally more inclined to try new food and change their food behaviour. Future studies may investigate what factors could be taken into account when communicating with adults, who are less likely to try new food. Moreover, all respondents were Italian students; therefore, a future comparison between cultures as variables influencing food choice motives and attitudes would provide a better understanding of the phenomenon. A further limitation stems from the type of questions focused on food choice in general, since producing and selling terrestrial invertebrates as novel food is still prohibited in Italy. Nevertheless, if novel food enters in the Italian market, future research might focus on specific food choice motives.

Study 2 was a laboratory experiment; thus, the setting is different from a naturalistic tasting or purchasing environment. Nevertheless, for our purpose, the priority was a high level of control offered by a laboratory that can guarantee a valid study. To avoid as much as possible the potential alteration, all the neuropsychophysiological signals were measured in a relaxed and neutral condition. This provides a benchmark of each subject’s activation in the specific experimental situation to measure the emotional activation due to each specific stimulus. A second limitation is the use of a specific invertebrate: earthworms. In order to compare different communication strategies, the use of more types of novel food would have introduced other intervenient variables, leading to a more difficult interpretation of the unique effects of the food labels. In order to reach more generalizable results, future research should try to replicate the same experiment with different types of meal (e.g., cricket meal) and food products (e.g., pastries). Moreover, we considered only young consumers with a high educational level because they could be the future consumers of this type of novel food. This constitutes just a starting point and further research could deepen the study considering older populations and people with different educational levels. In addition to these limitations, there is another issue: the sample size. Because of the small size of the sample, the main problem is the interpretation of results. Despite the fact that neuromarketing research using neurophysiological data usually has a small sample size [52], future research should have a larger sample size, in order to guarantee a higher reliability. Finally, our study focused on the food labels claim; future research should study in greater depth the effect of the visual elements on the label (e.g., pictures of earthworms/flour).

## Figures and Tables

**Table 1:** The modified Food Choice Questionnaire (FCQ), from Steptoe and colleagues (1995).

FCQ Item	Mean/SD	Cronbach’s Alpha	Standardised Factor Loading
*Health*	5.38/0.96	0.84	
Contains a lot of vitamins and minerals	5.31/1.36		0.84
Keeps me healthy	6.16/1.08		0.71
Is nutritious	5.92/1.08		0.70
Is high in protein	4.68/1.39		0.67
Is good for my skin/teeth/hair/nails etc.	4.69/1.74		0.67
Is high in fibres and roughage	4.88/1.46		0.74
Provides all the nutrients required	6.03/1.14		0.73
*Mood*	5.37/0.96	0.78	
Helps me cope with stress	5.09/1.57		0.80
Helps me cope with life	5.88/1.18		0.63
Helps me relax	4.45/1.60		0.71
Keeps me awake/alert	5.61/1.28		0.71
Cheers me up	5.01/1.58		0.71
Makes me feel good	6.22/1.03		0.61
*Convenience*	4.33/1.18	0.87	
Is easy to prepare	4.12/1.61		0.88
Can be cooked very simply	4.64/1.54		0.87
Takes no time to prepare	4.22/1.57		0.92
*Availability*	5.37/0.96	0.66	
Can be bought in shops close to where I live or work	5.35/1.42		0.86
Is easily available in shops and supermarkets	5.37/1.30		0.86
*Sensory appeal*	5.53/0.95	0.68	
Smells nice	5.31/1.43		0.78
Looks nice	4.75/1.61		0.75
Has a pleasant texture	5.61/1.30		0.75
Tastes good	6.48/0.86		0.58
*Natural content*	5.09/1.36	0.81	
Contains no additives	4.85/1.65		0.84
Contains natural ingredients	5.48/1.47		0.85
Contains no artificial ingredients	4.95/1.67		0.87
*Price*	4.74/1.34	0.80	
Is not expensive	4.79/1.47		0.91
Is cheap	4.68/1.47		0.91
*Weight control*	4.17/1.38	0.80	
Is low in calories	3.62/1.57		0.88
Helps me control my weight	4.81/1.71		0.80
Is low in fat	4.08/1.59		0.86
*Familiarity*	3.92/1.28	0.70	
Is what I usually eat	3.48/1.59		0.81
Is familiar	4.48/1.60		0.82
Is a well-known brand	3.44/1.68		0.73
*Ethical concern*	4.63/1.13	0.75	
Has a food quality certification (PDO, PGI or TSG)	4.79/1.61		0.56
Is organic	4.10/1.69		0.76
Is produced/packaged in an environmentally friendly way	5.67/1.47		0.70
Is locally produced/cultivated	4.79/1.60		0.73
Is a fair-trade product	3.96/1.64		0.77
